# Host clinical preceptors’ perceptions of professionalism among learners completing global health electives

**DOI:** 10.5116/ijme.5b40.6e4b

**Published:** 2018-07-27

**Authors:** Chris A. Rees, Elizabeth M. Keating, Heather Lukolyo, Padma Swamy, Teri L. Turner, Stephanie Marton, Jill Sanders, Edith Q. Mohapi, Peter N. Kazembe, Gordon E. Schutze

**Affiliations:** 1 Division of Emergency Medicine, Boston Children's Hospital, Harvard Medical School, USA; 2 University of Utah, Department of Pediatric Emergency Medicine, Salt Lake City, UT, USA; 3 Department of Pediatrics, Baylor College of Medicine, Houston, TX, USA; 4 Baylor College of Medicine Children's Foundation Lesotho, Maseru, Lesotho; 5 Baylor College of Medicine Children's Foundation Malawi, Lilongwe, Malawi

**Keywords:** Professionalism, global health, medical education, host perspective, sub-Saharan Africa

## Abstract

**Objectives:**

This study aims to gain an understanding of the perceptions of host clinical preceptors in Malawi and Lesotho of the professionalism exhibited by short-term learners from the United States and Canada during short-term global health electives.

**Methods:**

Focus group discussions were conducted with 11 host clinical preceptors at two outpatient pediatric HIV clinics in sub-Saharan Africa (Malawi and Lesotho). These clinics host approximately 50 short-term global health learners from the United States and Canada each year. Focus group moderators used open-ended discussion guides to explore host clinical preceptors’ perceptions of the professionalism of short-term global health learners. Thematic analysis with an inductive approach was used to identify salient themes from these focus group discussions.

**Results:**

Eleven of the 18 possible respondents participated in two focus group discussions. Adaptability, eagerness to learn, active listening, gratitude, initiative, and punctuality was cited as professional behaviors among short-term global health learners. Cited unprofessional behaviors included disregard of local clinicians’ expertise and unresponsiveness to feedback. Host clinical preceptors described difficulty providing feedback to short-term global health learners and discrepancies between what may be considered professional in their home setting versus in the study settings. Respondents requested pre-departure orientation for learners and their own orientation before hosting learners.

**Conclusions:**

Both host clinical preceptors and short-term global health learners should be aware that behaviors that may be considered best practice in one clinical setting may be perceived as unprofessional in another. Future studies to develop a common definition of professionalism during short-term global health electives are merited.

## Introduction

Nearly one-third of medical students in the United States complete rotations in countries other than their own during medical school,^1^ and one-fifth of pediatrics residents spend time abroad during their residency training.^2^ These rotations, which typically last up to six weeks, are called short-term global health electives (STGHEs). Participation in STGHEs is associated with improved cross-cultural communication, enhanced knowledge of tropical diseases, and decreased reliance on laboratory testing and imaging among medical trainees when they return to their high-resource setting.^3^^-^^5^

Assessing medical learners’ professionalism while they are in their home setting is a routine part of many medical curricula in high-resource settings.^6^ However, formal descriptions of the assessment, and definition, of professionalism of medical learners from high-resource settings during STGHEs are lacking. Although the term professionalism encompasses many elements, its framework includes the pillars of excellence, humanism, accountability, and altruism based on ethical and legal understanding, communication skills, and clinical competence.^7^ Professionalism in clinical settings is a complex social construct and includes both workplace-based norms as well as the health care delivery system.^8^ Thus, appropriate behavior in one setting may be viewed as unprofessional in another due to differing cultural norms.

Despite the popularity of STGHEs among medical learners from high-resource settings and the importance placed on professionalism in medical education, few studies have investigated host clinical preceptors’ perceptions of professionalism exhibited by medical learners’ during STGHEs.^9^^,^^10^ These previous studies have used pre-determined definitions of professionalism despite cultural and contextual differences between resource-rich and resource-limited clinical settings. Understanding host clinical preceptors’ expectations of medical learners’ professionalism during STGHEs is a crucial step in improving the quality of STGHEs as it will allow program directors to better prepare medical learners for potentially different professional expectations in a culture outside of their own. The objective of this study was to explore host clinical preceptors’ perceptions of medical learners’ professionalism during STGHEs at two clinical sites in sub-Saharan Africa and to gain further understanding of methods to improve STGHEs for both rotators and preceptors.

## Methods

### Study design and participants

The authors employed a qualitative study design drawing upon constructivist grounded theory, which emphasizes the ways in which meaning is created socially and experientially.^11^ The authors selected focus group discussions to facilitate an open discussion between study participants. The authors developed a semi-structured discussion guide based on a review of the existing literature on professionalism in medical education, using an iterative process until authors achieved consensus on the content of the discussion guide. Discussion guides favored open-ended questions designed to elicit experiential data. Careful attention was paid so that questions and discussion probes were worded neutrally. All participants provided written informed consent to participate in the study. The only incentive provided was refreshments for each participant during the focus group discussion. Demographic questionnaires were completed by participants at the beginning of each focus group discussion. Moderators conducted focus group discussions in English.

This study was conducted at two Baylor College of Medicine Clinical Centers of Excellence in sub-Saharan Africa (Malawi and Lesotho). Annually, these sites host approximately 50 STGHE learners from multiple institutions in the United States and Canada. These sites were chosen because of the large volume of STGHE learners and because several of the authors had long-standing working relationships with these sites. STGHE learners include medical students, residents, and fellows who spend up to six weeks at these outpatient sites. These sites are part of the Baylor International Pediatric AIDS Initiative, which provides family-centered health care, health professional training for both local and international learners, and clinical research focused on HIV/AIDS, tuberculosis, malaria, malnutrition, and other conditions impacting the health and well-being of children and families worldwide.^12^

**Figure 1:**
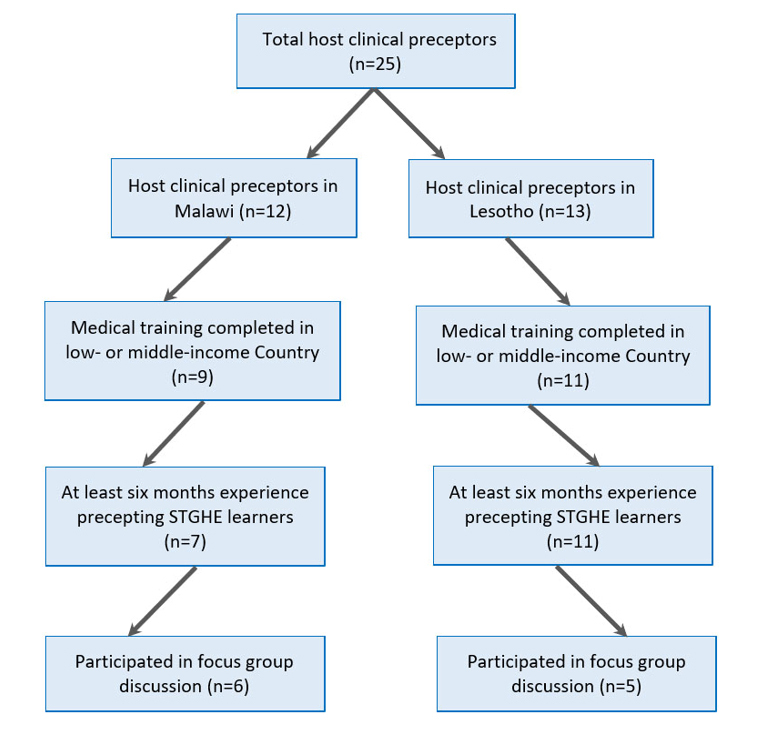
Host clinical preceptors meeting inclusion criteria for focus group discussions on professionalism among learners during short-term global health electives (STGHE)

The authors used purposive sampling to identify potential participants by obtaining a list of all employees who work as host clinical preceptors. Host clinical preceptors in this study were clinicians in Malawi and Lesotho who supervise the clinical care delivered by medical learners from the United States and Canada during STGHEs. Host clinical preceptors were invited by email and by announcement at staff meetings to participate in the study if they met all of the following inclusion criteria: employees at the Clinical Centers of Excellence in either Malawi or Lesotho, had at least six months of experience supervising STGHE learners, and completed their medical training in a low- or middle-income country. Host clinical preceptors were pediatricians, clinical officers (i.e., clinicians who had three years of medical education in Malawi after the high school equivalent and two years after the high school equivalent in Lesotho), and medical officers (general practitioner physicians who have completed medical school and internship but no residency or sub-specialty training). All host clinical preceptors at the two sites were fluent in English. Host clinical preceptors were excluded if they did not meet the study’s inclusion criteria. Six host clinical preceptors participated in the focus group discussion in Malawi and five in Lesotho (Figure 1), representing 85.7% and 45.5% of all host clinical preceptors in the respective countries who met inclusion criteria. Host clinical preceptor demographics are found in Table 1.

### Data collection methods

To establish the credibility of findings, moderators trained in focus group methodology (authors CAR and EMK) conducted one focus group discussion at each of the two sites (Malawi, April 2016 and Lesotho, June 2016), with the co-moderators present to assist with logistics. The moderators and co-moderators were physicians from the United States who had each been placed in their respective site for a 12-month clinical assignment who worked alongside focus group participants. Each focus group discussion was held in a private conference room in each of the clinics. At the beginning of the session, the moderators introduced themselves and presented the rationale behind the study. Each focus group lasted between 60-90 minutes and questions were probed until saturation was achieved. To establish confirmability of the findings and to make the data available for review by other investigators, interview moderators audio-recorded and manually transcribed interviews and double-checked transcripts for accuracy. Moderators de-identified data at the time of transcription. Transcripts were uploaded into Dedoose version 7.5.17 (Los Angeles, California), a secure, cloud-based qualitative analysis software.^13^

The study protocol was approved by the Malawi National Health Sciences Research Committee, the Lesotho Ministry of Health National Health Research Ethics Committee, and the Institutional Review Board of Baylor College of Medicine.

**Table 1:** Participant demographics in focus group discussions on professionalism among learners during short-term global health electives (STGHEs) (N=11)

Demographic		Malawi (n)	Lesotho (n)	Total (n)	%
Gender	Female	2	5	7	63.6
	Male	4	0	4	36.4
Nationality	Malawian	6	0	6	54.5
	Mosotho (Lesotho)	0	3	3	27.3
	Myanmar	0	1	1	9.1
	Nigerian	0	1	1	9.1
Country of medical training	Malawi	6	1	7	63.6
	Myanmar	0	1	1	9.1
	Nigeria	0	1	1	9.1
	South Africa	0	2	2	18.2
Years in clinical practice	2-5	1	0	1	9.1
	6-10	3	4	7	63.6
	>10	2	1	3	27.3
Role at center of excellence	Clinical Officer	6	0	6	54.5
	Medical Officer	0	4	4	36.4
	Pediatrician	0	1	1	9.1
Hours per month spent supervising STGHE learners	0-20	0	3	3	27.3
	21-40	4	2	6	54.5
	41-60	2	0	2	18.2
Number of STGHE learners supervised in past 12 months	<5	1	1	2	18.2
	5-10	4	4	8	72.7
	11-15	1	0	1	9.1

### Data analysis

Two authors experienced in qualitative research who did not participate in the moderation of focus groups (HL and PS) performed a thematic analysis to explore salient topics that emerged during the focus group discussions. To establish the dependability of the findings, two authors (HL and PS) independently coded the transcripts using an inductive approach. After independent coding, the two authors discussed disagreements with the larger research team and used a consensus-building process to identify codes that most accurately and completely represented the data. The authors also informally assessed the wording of questions and probes as captured in transcripts to ensure there were no leading questions. Finally, the authors discussed overarching categories, themes, and representative quotations.

## Results

Analysis of the transcripts from these focus group discussions revealed several themes as defined in Table 2.

### Perceived professional behaviors

Host clinical preceptors cited several attributes and behaviors among STGHE learners as professional. These attributes included adaptability and initiative. Perceived professional behaviors included active listening, gratitude, and punctuality. One host clinical preceptor commented:

“You will find that some learners are ready to learn. They are polite. They are always on time, and they are ready to listen to you.” (No. 4, Male, Malawian)

Another host clinical preceptor commented on the added benefit of exposure to new resources and information while hosting STGHE learners:

“Some learners are very interested in the patients, and they provide their input when you are seeing patients together. They even suggest resources where you can get more information.” (No. 7, Female, Mosotho)

Several host clinical preceptors commented on the empathetic attributes of STGHE learners. One preceptor remarked:

“At times, you think you are done with the patient encounter and the learner begins to talk to the caregiver about what the family is going through. They are very empathetic.” (No. 2, Male, Malawian)

**Table 2:** Themes that emerged during focus group discussions on professionalism among learners during short-term global health electives (STGHE) in Malawi and Lesotho

Theme	Definition
Perceived professional behaviors	Behaviors exhibited by STGHE learners that host clinical preceptors in Malawi and Lesotho felt were professional in the clinical setting
Perceived unprofessional behaviors	Behaviors exhibited by STGHE learners that host clinical preceptors felt were unprofessional in the clinical setting
Consequences of unprofessional behaviors	Both intended and unintended consequences of STGHE learners’ unprofessional behaviors from the perspective of host clinical preceptors
Influence of cultural context on professionalism	How differing cultural backgrounds of STGHE learners and host clinical preceptors contribute to perceived professional and unprofessional behaviors of STGHE learners
Proposed solutions to improve professional behaviors among STGHE learners	Ideas as proposed by host clinical preceptors that may lead to improved professionalism among STGHE learners during global away rotations

### Perceived unprofessional behaviors

It was perceived that learners experience difficulty adapting to the medical management in Malawi and Lesotho. Host clinical preceptors perceived that STGHE learners’ form of communication differed from that which is customary in the study settings. When disagreements arose regarding how a patient should be managed, many host clinical preceptors perceived that learners displayed negative attitudes, superiority, inflexibility, or authoritarianism:

“At times, I do not agree with the management that the learners propose, but I still try to listen to their input. There are many times that they are resistant to the management that I propose.” (No. 9, Female, Mosotho)

Moreover, cited unprofessional attributes and behaviors included unresponsiveness to feedback, apathy, and inappropriate interruptions. Host clinical preceptors also cited unprofessional dress including informal clothing or wearing “revealing clothing such as miniskirts” as an unwanted behavior among STGHE learners (No. 10, Female, Mosotho). Disregard for local clinical expertise, especially for health care workers that were not physicians, was commonly cited as an unprofessional behavior among STHGE learners. One host clinical preceptor stated:

“It becomes very difficult to impart knowledge on some learners because, as the saying that goes, 'You can take a horse to the well, but you can’t force it to drink.' So, they may be with you physically, but their mind is elsewhere.” (No. 4, Male, Malawian)

### Consequences of unprofessional behaviors

Host clinical preceptors stated that when STGHE learners act unprofessionally, they affect the perception of all STGHE learners. In some cases, unprofessional interactions may make host clinical preceptors hesitant to precept STGHE learners in the future. Moreover, some host clinical preceptors perceived that the pace and quality of patient care were negatively affected when STGHE learners act unprofessionally:

“Let’s say that I am sitting in the room with a learner who has annoyed me; it means that, let’s say in a day I see 15 patients, that particular day I won’t see 15. Not even close." (No. 5, Male, Malawian)

Some participants felt that vocal disagreements between host clinical preceptors and learners could undermine the patient-clinician relationship:

“If a patient sees you and the learner argue then they may not end up trusting you. That is dangerous in the eyes of the patient. It could lead to mismanagement of our patients.” (No. 8, Female, Mosotho)

### The influence of cultural context on professionalism

Respondents described discrepancies between what may be considered professional in the United States but not in settings similar to the two sites in sub-Saharan Africa, citing differing communication styles with both colleagues and patients:

“It seems to be a problem in the West if you don’t explain to patients what they are suffering from. But here it doesn’t matter unless the patient or the guardian asks about it. That’s when you go into the details.” (No. 1, Male, Malawian)

Host clinical preceptors also cited cultural differences as a potential challenge in providing feedback to STGHE learners.

“When the feedback is troublesome it is difficult to say. We tend to think, ‘Oh, they will only be here for a month. I can live with that for a month.’ So, we don’t give negative feedback. We also don’t know how they will respond to our feedback.” (No. 11, Female, Nigerian)

### Proposed solutions to enhance both patient care and professional behaviors among STGHE learners in a different cultural context

Host clinical preceptors requested pre-departure preparation for STGHE learners with a clear explanation of host clinical preceptors’ expertise, learners’ role, and professionalism across cultures. Respondents requested an orientation before hosting learners with clear descriptions of the learner’s level of training.

“If we are told that these are medical doctors from the United States but you find out that the person is a medical student, it is a bit confusing. It reflects a lack of truth in it.” (No. 9, Female, Mosotho)

Host clinical preceptors also requested the creation of a formal mechanism for two-way feedback:

"Sitting down with learners to fill out evaluations may be difficult because we may also ‘swallow it’. And it should be both ways, we can evaluate them and give formal feedback and they can also give formal feedback about us.” (No. 3, Female, Malawian)

Several respondents requested improved communication between sending and hosting institutions. One such example was the following quote:

“There should be somebody that we can talk to when we have feedback. We should be able to notify them of issues so that learners do not repeat the same mistakes. This way learners will know what is expected of them based on previous experiences.” (No. 10, Female, Mosotho)

## Discussion

Host clinical preceptors from the two STGHE sites included in this study cited many professional behaviors that STGHE learners exhibit during their time overseas. Similar to the American Board of Pediatrics’ list of eight components of professionalism (honesty/integrity; reliability/responsibility; respect for others; compassion/empathy; self-improvement; self-awareness/knowledge of limits; communication and collaboration; and altruism and advocacy),^14^ host clinical preceptors cited initiative, reliability, and eagerness to learn.

Host clinical preceptors also cited gratitude and active listening as components of professionalism, which are not included by the American Board of Pediatrics. The development of components of professionalism specific to STGHEs may reduce the perceived divide between professional behaviors in the United States and sites like Malawi and Lesotho.

Host clinical preceptors commented on multiple unprofessional behaviors that STGHE learners exhibit, some of which are similar to those encountered in traditional medical education settings in the United States and Canada. Other studies assessing host clinical preceptor perceptions of STGHE learners have similarly found learners’ hesitancy and apathy as common unprofessional behaviors.^10^ Moreover, similar to previous studies,^15^ host clinical preceptors in this study cited disrespect for health workers as problematic behavior exhibited by STGHE learners. Our study adds to these findings by showing how unprofessional behaviors negatively affect the host clinical preceptor-learner relationship both by threatening the patient-clinician relationship and shedding light on preceptor’s hesitation to precept subsequent learners.

Our study demonstrates how context influences perceived professionalism among STGHE learners. This aligns with previous studies that have described how regional and cultural perspectives influence perceived professionalism.^16^^,^^17^ A study conducted in another sub-Saharan African country questioned the “universal applicability of internationally accepted standards of professionalism” due to differing cultural norms and resource availability.^18^ Several studies conducted in the United States have stated that defining professionalism is in need of development and clarity.^19^^,^^20^ Though defining professionalism is a point of debate, particularly across different cultural settings, professionalism should be relevant to local social and cultural norms.^21^ Our study shows a key difference in the perceived need to communicate with patients and families at these two sites in sub-Saharan Africa. Medical students and residents who are trained in the United States are taught that effective communication with patients improves both patient satisfaction and outcomes.^22^^,^^23^ The host clinical preceptors’ perception of not needing to explain a patient’s condition to the family may represent a lack of professionalism on the host clinical preceptor’s part. Differing views of professionalism in both STGHE learners and host clinical preceptors may contribute to the divide in the perception of professionalism.

Host clinical preceptors also cited difficulty in providing feedback given the different perceptions of professionalism. Appropriately structured feedback significantly benefits medical learners’ achievement and growth.^24^ Although giving feedback was cited as a challenge for host clinical preceptors, a common theme was the desire to receiving feedback about the experience of STGHE learners. The creation of a mechanism for bidirectional feedback may allow for STGHEs to move toward mutual benefit.

Lastly, host clinical preceptors provided their insight on how orientation, proper introductions, and role definitions may improve professionalism among STGHE learners. Similarly, other studies have cited professionalism as a key component that should be included in pre-departure training for STGHE learners.^25^ Other studies assessing host clinical preceptors’ perceptions of international visiting faculty have called for “orientation to clinical work in the host’s setting”.^26^ Future training programs aimed at improving professionalism among STGHE learners working in different clinical and cultural settings may employ a case-based approach,^27^ reflections,^28^ or using simulated patients in clinical scenarios,^29^ as previous studies have shown these to be effective methods in teaching principles of professionalism. Faculty from sending institutions should be aware of differing perceptions of professionalism during STGHEs and strive to prepare learners for these differences prior to their departure.

This study is subject to several limitations. There was a lower response rate in Lesotho than there was in Malawi as many preceptors in Lesotho go on outreach and work at other sites each day. However, the responses from Lesotho were very similar to Malawi. Moreover, we were unable to obtain demographic data of the non-responder group so it is unclear what selection bias may have been introduced by the participants who were available for the focus group discussions. This study was conducted at two sites in two specific countries in sub-Saharan Africa, making the transferability to other parts of the world where STGHEs are common difficult. This study did not differentiate between medical students and pediatric residents when posing questions to host clinical preceptors as it is often difficult for host clinical preceptors to recall the precise level of training of individual learners. Also, the focus group moderators and co-moderators were physicians who were trained in the United States which may have introduced bias in regard to their own perceptions of professionalism in the clinical setting, though all had been living and working at the respective clinical sites for several months at the time of the study. Lastly, as this study was conducted only with host clinical preceptors, there was not an attempt to capture the STGHE learners’ perspective on how professionalism may differ at the two STGHE sites in comparison to their home institutions.

## Conclusions

Host clinical preceptors perceive that STGHE learners exhibit a spectrum of attributes and behaviors, ranging from those perceived to be unprofessional to professional. Some of these perceived unprofessional attributes and behaviors are dependent upon the local context. Exhibiting professionalism within a different context is a necessary skill for all learners, but particularly for those who deliver healthcare outside the primary system in which they train. Both host clinical preceptors and short-term global health learners should be aware that behaviors that may be considered best practice in one clinical setting may be perceived as unprofessional in another. Future studies to develop a common definition of professionalism during short-term global health electives are merited.

### Acknowledgements

We would like to thank the host clinical preceptors who participated in this study and provided their valuable insight to improve short-term global health electives. We would also like to thank Drs David S. Sullivan and Kimberley Farr for their assistance in moderating the focus group discussions in this study.

### Conflict of Interest

The authors declare that they have no conflict of interest.
